# Human Amnion Epithelial Cells Impair T Cell Proliferation: The Role of HLA-G and HLA-E Molecules

**DOI:** 10.3390/cells9092123

**Published:** 2020-09-19

**Authors:** Fabio Morandi, Danilo Marimpietri, Andre Görgens, Alessia Gallo, Raghuraman Chittor Srinivasan, Samir El-Andaloussi, Roberto Gramignoli

**Affiliations:** 1 Stem Cell Laboratory and Cell Therapy Center, IRCCS Istituto Giannina Gaslini, Via Gaslini5, 16147 Genova, Italy; danilomarimpietri@gaslini.org; 2 Department of Laboratory Medicine, Division of Biomolecular and Cellular Medicine, Karolinska Institutet, 14157 Stockholm, Sweden; andre.gorgens@ki.se (A.G.); samir.el-andaloussi@ki.se (S.E.-A.); 3 Institute for Transfusion Medicine, University Hospital Essen, University of Duisburg-Essen, 45147 Essen, Germany; 4 Department of Research, IRCCS ISMETT (Istituto Mediterraneo per i Trapianti e Terapie ad alta specializzazione), Via E.Tricomi 5, 90127 Palermo, Italy; agallo@ismett.edu; 5 Department of Laboratory Medicine, Division of Pathology, Karolinska Institutet, Alfred Nobels Alle 8, Huddinge SE-141 83, 14157 Stockholm, Sweden; ragsyrazor@gmail.com

**Keywords:** amnion epithelial cells, immunomodulation, HLA-G, perinatal stem cells, extracellular vesicles

## Abstract

The immunoprivilege status characteristic of human amnion epithelial cells (hAECs) has been recently highlighted in the context of xenogenic transplantation. However, the mechanism(s) involved in such regulatory functions have been so far only partially been clarified. Here, we have analyzed the expression of HLA-Ib molecules in isolated hAEC obtained from full term placentae. Moreover, we asked whether these molecules are involved in the immunoregulatory functions of hAEC. Human amnion-derived cells expressed surface HLA-G and HLA-F at high levels, whereas the commonly expressed HLA-E molecule has been measured at a very low level or null on freshly isolated cells. HLA-Ib molecules can be expressed as membrane-bound and soluble forms, and in all hAEC batches analyzed we measured high levels of sHLA-G and sHLA-E when hAEC were maintained in culture, and such a release was time-dependent. Moreover, HLA-G was present in extracellular vesicles (EVs) released by hAEC. hAEC suppressed T cell proliferation in vitro at different hAEC:T cell ratios, as previously reported. Moreover, inhibition of T cell proliferation was partially reverted by pretreating hAEC with anti-HLA-G, anti-HLA-E and anti-β2 microglobulin, thus suggesting that HLA-G and -E molecules are involved in hAEC-mediated suppression of T cell proliferation. Finally, either large-size EV (lsEV) or small-size EV (ssEV) derived from hAEC significantly modulated T-cell proliferation. In conclusion, we have here characterized one of the mechanism(s) underlying immunomodulatory functions of hAEC, related to the expression and release of HLA-Ib molecules.

## 1. Introduction

The human placenta is a complex organ, whose role in regenerative medicine applications have been largely proposed and is gaining interest worldwide. Placenta tissue is composed of both maternal and fetal tissues, with the latest as a source of multipotent stem cells successfully employed in innovative biomedical approaches [1]. The fetal membrane in direct contact with the fetus (amnion membrane) is composed of cuboidal epithelial cells firmly adherent to a thick basement membrane, and an abundant extracellular matrix with intersperse fibroblast-like cells.

Human amnion epithelial cells (hAECs) can be released from the amnion membrane and depicted by epithelial characteristics and membrane antigens. While expressing high levels of classic epithelial adhesion molecules, such as EpCAM (CD326) or integrin subunits (including CD29 and CD49f), hAEC tested negative for stromal cell markers [1,2]. Distinctive is the surface expression of embryonic stem cell markers, such as globoseries glycolipids (SSEA-3 and -4) and keratansulfate associated antigens (TRA 1-60 and 1-81) in hAEC [3,4,5]. AECs have been proved safe and non-tumorigenic upon transplantation, with a normal karyotype, lack of telomerase and limited growing potential in culture [5,6]. These features differentiate hAECs from pluripotent stem cells and support their use in cell-based therapies. As a result, recent studies have reported on the therapeutic potential of amnion-derived cells. Amnion epithelial cells have shown efficacy in different clinical and preclinical trials, representing a promising therapeutic approach in life-threatening models of inborn error of diseases and respiratory distress [7,8,9,10,11].

Amnion-derived epithelial cells display a characteristic expression of the non-canonical HLA class Ib molecule HLA-G, along with a low expression of HLA class Ia molecules, and deprival of HLA class II molecules [12,13,14,15]. These properties represent a physiological immunomodulatory mechanism characteristic of resident cells at the fetal–maternal interface.

HLA-G has been originally described as an important tolerogenic molecule expressed by cytotrophoblast cells at the maternal/fetal interface, to prevent immune recognition of (semi) allogeneic fetal tissue by maternal immune cells [16]. HLA-G belongs to ‘‘non-canonical” HLA-class Ib molecules, along with HLA-E, -F and -H [17]. In contrast to highly polymorphic HLA-class Ia molecules (HLA-A, -B and -C), HLA-Ib molecules display a scarce polymorphism, with a small number of alleles encoding a limited number of proteins, conveying immune responses in both physiological and pathological conditions [18]. HLA-G has been identified on mature human epithelial cells [19,20,21], including umbilical cord lining epithelial cells [22] and amnion/chorion epithelial and stromal cells [12,13,14,23,24,25], and its expression can be modulated in pathological settings [23,26,27]. HLA-G restraining effect may modulate the recipient immune response and induce tolerance to the host graft, as a therapeutic agent in (tissue or cell) transplantation [25]. The HLA-G molecule can be present in seven isoforms, four membrane-bound (m) isoforms (mHLA-G: HLA-G1, -G2, -G3 and -G4) and three soluble (s) isoforms (sHLA-G: HLA-G5, -G6 and -G7). In addition, sHLA-G1 is generated by shedding of the membrane-bound HLA-G1 isoform through metalloproteases-mediated cleavage [28]. HLA-G1/G5 isoforms are the most expressed and the most relevant in relation to immunomodulatory functions [29]. Both forms have been described as disulfide-linked dimers, which are more biologically active than monomers [30].

The constitutive presence of mHLA-G on the full-term amnion membrane and isolated hAEC has been shown [13], even after cryogenic preservation [12,23]. Conversely, to the best of our knowledge, no previous report described expression of HLA-E and HLA-F on hAEC. The HLA-E gene is expressed by all nucleated cells. However, HLA-E expression on the cell surface reflects cell activation and is in relation with interaction molecules as β2 microglobulin or nonapeptides derived from other HLA-I molecules, or viruses and other pathogens [31]. HLA-E may interact with the CD94/NKG2A inhibitory receptor, triggering immunosuppressive signals to NK and CD8 T cells, or with the activating CD94/NKG2C receptor, the hinge on the peptide bound to the HLA-E molecule [32]. Similarly, HLA-F surface expression is also triggered by cell activation, while gene expression is normally detected within cells. The HLA-F molecule can be expressed either associated with β2 microglobulin and peptides or as an open conformer (represented by heavy chain alone). These two different forms can bind to different receptors, triggering activating or inhibitory signals [33].

Few studies have previously described hAEC functions in relation to the release of extracellular vesicles (EVs) [34,35,36]. EV include microvesicles, exosomes and apoptotic bodies [37]. Microvesicles differ from exosomes in diameter (range 50–1000 nm and 40–120 nm, respectively) and genesis. Indeed, exosomes originate through an endocytic pathway, while microvesicles are released from the plasma membrane of activated or neoplastic cells. Among other surface molecules, microvesicles express integrins, selectins, CD40 or CD81, and are involved in intercellular communications, being captured by target cells through endocytosis, receptor-mediated endocytosis or fusion with the plasma membrane [38,39]. We have previously described that EVs released by tumor cells may carry some immunomodulatory molecules (i.e., adenosinergic ectoenzymes), contributing to the inhibition of the host antitumor immune response [40,41]. Moreover, several studies reported the role of HLA-G-bearing EV in modulation of an immune response in normal pregnancy or neoplastic context [42].

With such premises, the aim of our study was to assess whether HLA-Ib molecules (in particular HLA-G and HLA-E) are expressed and released by hAEC and may play a role in their immunomodulatory functions. Furthermore, we characterized the expression of HLA molecules on large-size (ls) and small-size (ss) EV derived from primary hAEC. Finally, the contribution of hAEC-derived ssEV and lsEV was assessed in relation to the modulation of the immune response.

## 2. Materials and Methods

### 2.1. Cell Preparations

Full term placentae from uncomplicated caesarean sections at a gestational age of 37–42 weeks were obtained from Karolinska University Hospital (Stockholm, Sweden) with Institutional Review Board approval. Isolation of hAEC was performed as previously described [1]. Briefly, the amnion membrane was surgically removed from the placenta surface and washed to remove blood cells. Epithelial cells were released by TrypLE (Thermo Fisher Scientific, Waltham, MA, USA) digestion (30 min) and immediately cryopreserved in Belzer solution supplemented with 10% dimethylsulfoxide (Sigma-Aldrich, St Louis, MO, USA) and stored in liquid nitrogen vapors. The cell viability range was 90–97% after isolation and 85–94% after cryopreservation, as assessed by the Trypan Blue exclusion test.

Peripheral blood samples were obtained from normal donors afferent at Blood Bank of Istituto Giannina Gaslini (Genoa, Italy). CD4^+^ T cells were isolated by incubating whole blood samples with RosetteSep^TM^ Human CD4^+^ T cells enrichment cocktail (StemCell Technologies, Cambridge, UK) following the manufacturer’s protocol. Unmanipulated CD4^+^ T cells were successfully obtained by Ficoll-Hypaque (Sigma) density gradient centrifugation.

### 2.2. Cell Culture

One week before experiments, one vial of frozen hAEC was thawed and 1.5 × 10^5^ cells were seeded in gelatin-coated plates. Cells were cultured at 37 °C in a humidified atmosphere of 5% CO_2_:95% air in the D-MEM medium supplemented with 10% fetal bovine serum, 1 mM non-essential amino acids, 4 mM L-glutamine, 55 µM2β-mercaptoethanol (all from Sigma-Aldrich) and 10 ng/mL recombinant human epidermal growth factor (rhEGF, Immunotools). Every two days, the medium was removed and fresh medium added. After 6 days of culture, hAEC were used for the experiments described below. For cell suspension experimental settings, hAEC were detached using Tripsin 0.05%/EDTA solution (Sigma-Aldrich).

### 2.3. Isolation of EV from hAEC

EVs were isolated from preconditioned supernatants obtained from different batches of hAEC, cultured as described above. EV were prepared by using two different approaches: to enrich lsEV, supernatants were collected after an additional 72 h of culture in D-MEM medium described above, supplemented with 10% EV-depleted fetal bovine serum. Supernatants were subjected to a differential centrifugation protocol as previously described [43]. Briefly, supernatants (10 mL) were centrifuged (3000× *g* for 15 min at 4 °C) to pellet large cell debris. The supernatant was collected in a suitable centrifugation tube and centrifuged (20,000× *g* for 1 h at 4 °C) in a fixed-angle rotor, washed once in PBS and resuspended in 50 µL of binding buffer (PBS containing 0.5% BSA and 2 mM EDTA; all purchased from Sigma Aldrich). lsEV size and polydispersity were analyzed using the Zetasizer Nano ZS90 particle sizer at a 90° fixed angle (Malvern Instruments, Worcestershire, UK), as previously described [44]. In some experiments, lsEV were isolated using a well-defined ultrafiltration/TFF method [45]. In those samples, particle size and concentration were determined via a nanoparticle tracking analysis (NTA) using NanoSight NS500 equipped with NTA 2.3 analytical software and a 488 nm laser, as previously described [45].

Small size EV were isolated from 10 mL of hAEC supernatant mixed with ExoQuick solutions and ExoQuick-TC™ polymers (System Biosciences, Palo Alto, CA, USA), according to the manufacturer’s protocol. Briefly, cell supernatants were centrifuged at 3000× *g* for 15 min to remove cells and cell debris. The supernatant was transferred to sterile vessels and mixed with ExoQuick solution/polymers, vortexed and stored at 4 °C for 30 min. Samples were centrifuged at 1500× *g* for 30 min at room temperature and the pellet suspended in nuclease-free water. For HLA-G staining ssEV were analyzed by flow cytometry after vesicle adsorption onto latex beads as previously reported [44].

hAEC-derived EV preparations were suspended in 100 µL of binding buffer or culture medium for subsequent experiments.

### 2.4. Inhibition of EV Release

In some experiments, hAEC were treated with the following inhibitors (all purchased from Sigma Aldrich): Manumycin A (10 µM) and GW4869 (1 µM, inhibitors of ssEV release) or D-Pantethine (1 mM, inhibitor of lsEV release). Cells were cultured in D-MEM medium described above, supplemented with 10% EV-depleted fetal bovine serum for additional 48 h in the presence of inhibitors before being detached and used for the cell proliferation assay. Supernatant was collected and subjected to 0.8 µM filtration (to remove cell debris) before being subjected to ultracentrifugation for EV isolation. To confirm inhibition of EV release, lsEV and ssEV concentration was analyzed using Zetasizer Nano ZS90, as described above.

### 2.5. Flow Cytometry

The presence of immunomodulatory molecules was detected on hAEC intact cells and hAEC-derived EV using the following monoclonal antibodies: FITC-conjugated anti-HLA-G (clone: MEM-G/9, Exbio), PE-conjugated anti-HLA-F (clone: 3D11, Biolegend) and purified anti-HLA-E (clone: MEM-E/02, Exbio). PE-conjugated rat anti-mouse IgG1 (Beckman Coulter) was used as a secondary reagent for anti-HLA-E mAb.

Cells were run on a Gallios cytometer and analyzed using Kaluza software version 1.1.11052.10190 (built on 7/9/2010, Beckman Coulter). Data are presented as the percentage of positive cells or the mean relative of fluorescence intensity (MRFI, for cells and EV), obtained as follows: mean fluorescence obtained with specific mAb normalized to mean fluorescence obtained with irrelevant isotype-matched mAb.

The multiplex-bead based analysis of surface markers was performed on ssEV using the MACSPlex Exosome kit (MiltenyiBiotec) by using allophycocyanin (APC)-conjugated pan-tetraspaninantibodies included in the kit for detection (CD9/CD63/CD81), as previously described [46]. In brief, ssEV were incubated with capture beads (input dose: 1 × 10^9^ EVs as estimated by NTA, diluted to a total volume of 120 µL with PBS), incubated overnight at room temperature for the capture step, and subsequently incubated with a mixture of pan-tetraspanin antibodies for 1 h followed by washing. The samples were analyzed with a MACSQuant Analyzer 10 flow cytometer (MiltenyiBiotec). Data are presented following the background subtraction of the median APC fluorescence intensity (MedFI) values for each bead population, i.e., values obtained for non-EV containing controls (beads + antibodies) were subtracted from sample values (beads + EVs + antibodies) for each bead population.

### 2.6. ELISA

Enzyme-linked immunosorbent assay (ELISA) specific for soluble HLA-G1/G5, HLA-G5 and HLA-E was performed as previously described [47]. Briefly, MaxiSorp Nunc-Immuno 96 microwell plates (Nunc A/S) were coated overnight at 4 °C with 1 µg/mL of MEM-G9 monoclonal antibody (specifically for sHLA-G1/G5; Exbio), 5A6G7 monoclonal antibody (specific for HLA-G5; Exbio) or 3D12 monoclonal antibody (specific for HLA-E; eBioscience). After washes with PBS 0.05% Tween 20 (washing buffer), plates were saturated with 200 μL/w of PBS 2% BSA (Sigma) for 30 min at RT. One hundred microliters of hAEC supernatants (samples) and serial dilutions of supernatants from the 721.221 cell line, transfected either with HLA-G1 or HLA-G5 (standard for HLA-G, kindly provided by Dr. Francesco Puppo, DIMI, Genoa) or total extract from normal peripheral blood mononuclear cells (standard for HLA-E) were added in the corresponding wells and plates were incubated at RT for 1 h. After washes, 100 μL of detection reagent (HRP-conjugated anti-β2 microglobulin mAb, Exbio) was added, and plates incubated for 1 h at RT. After washes, 100 μL of TMB (substrate for HRP, Sigma-Aldrich) was added, and the reaction was stopped approximately 30 min later by adding H_2_SO_4_ 5N. Absorbance at 450 nm was measured using Infinite^®^ 200 PRO spectrometer (Tecan Group Ltd.). Results are expressed as ng/mL (sHLA-G1/G5 and sHLA-G5) or arbitrary units (U)/mL (sHLA-E). One arbitrary unit is equal to the quantity of sHLA-E in 1 µg of the total normal peripheral blood mononuclear cells extract.

### 2.7. Cell Proliferation Assay

Cell proliferation was assessed using the carboxyfluoresceindiacetatesuccinimidyl ester (CFSE) dilution assay. Briefly, CD4^+^ T cells were stained with CFSE (Invitrogen, 1 µg/mL, 15 min at 37 °C), washed and cultured in RPMI medium supplemented with fetal bovine serum at 37 °C and 5% CO_2_. Subsequently, T cells were seeded in 96 flat-bottom well plates (Costar Corning, 100,000/well) and stimulated with anti-CD3/anti-CD28 mAb coated beads (T cell activation/expansion kit, Miltenyi Biotec), in the presence or absence of hAEC (at hAEC:effector cell ratios ranging from 1:1 to 1:8). In the control group, hAEC were treated for 30 min with anti-HLA-G (#87G, Exbio, 10 µg/mL), anti-HLA-E (#3D12, Biolegend, 20 µg/mL) or anti-β2 microglobulin (kindly supplied by Dr. Fabio Malavasi, University of Turin, Italy).

In another set of experiments, 100,000 stimulated T cells were cultured in the presence of ssEV or lsEV derived from 40 mL of hAEC supernatant derived from 75 cm^2^ at confluence (5 millions of cells) and resuspended in 100 µL of culture medium, or with hAEC (at hAEC:effector cell ratio 1:1) previously cultured in the presence of inhibitors of EV release, as described above.

Effector cells were harvested after 6 days, and stained with PE-conjugated anti-CD4 mAb (Beckman Coulter). CFSE/CD4 positive cells were analyzed using the Gallios cytometer and Kaluza software.

### 2.8. Gene Profiling by qPCR

Thawed hAEC were lysed in Trizol™ solution (Life Tech, Carlsbad, CA, USA) and total RNA was isolated according to the manufacturer’s instructions. Total RNA was converted to complementary DNA using the high capacity cDNA kit (Life tech, Carlsbad, CA, USA). Gene expression was assessed using TaqMan assays for HLA-G (HS00365950), HLA-E (HS03045171) and HLA-F (HS04185703). Reactions were run in duplicate with human cyclophilin A (PPIA) (Hs99999904_m1) as a house keeping gene as the control for all experiments. Calculation of the relative levels of expression were done according to the comparative Ct-method as follows: 2^(−ΔCt)^, where ΔCt = (*Ct* gene of interest –*Ct* internal control Cyclophilin). *Ct* values for the gene of interest 35 or higher were considered as unreliable and ignored from the calculation.

### 2.9. Statistical Analysis

Statistical analysis was performed using Prism 5.03 software (GraphPad Software). Gaussian distribution of data was analyzed using the Kolmogorov–Smirnov test. The Student’s *t* test or Mann–Whitney test was used to compare data, depending on data distribution. The significance range is represented as follows: * *p* < 0.05 (significant), ** *p* < 0.005 and *** *p* < 0.0005.

## 3. Results

### 3.1. Human Amnion Epithelial Cells Express and Release HLA-Ib Molecules

**Figure 1:**
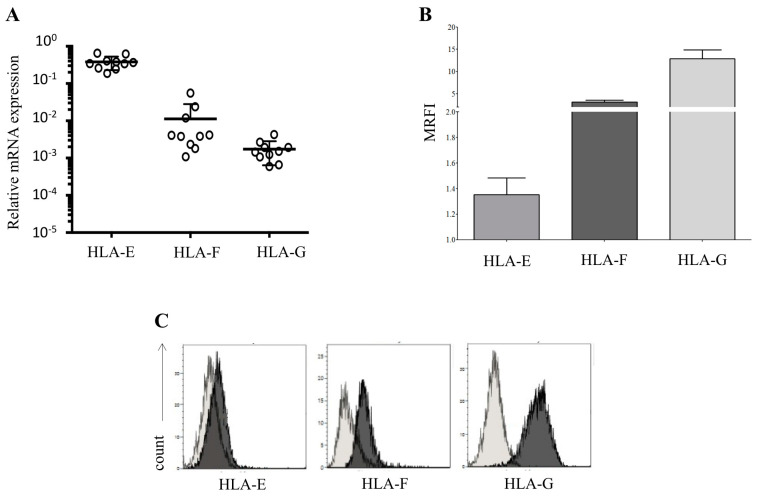
Expression of HLA-class Ib molecules by human amnion epithelial cells (hAECs). (**A**) Transcriptional analysis on 10 different batches of hAEC for all three HLA-Ib molecules; (**B**) the expression of immunomodulatory molecules HLA-E, HLA-F and HLA-G was assessed by flow cytometry on 17 different batches of hAEC. Data are expressed as mean relative of fluorescence intensity (MRFI) ± SE; (**C**) Panel C shows a representative staining. Light grey profiles indicate staining with isotype control, whereas dark grey profiles indicate staining with specific mAbs.

First, we evaluated the expression of HLA molecules on hAECs derived from different donors. We analyzed 10 batches of hAEC products isolated from different donors, and we consistently detected expression of HLA-E, HLA-F and HLA-G at the RNA level (Figure 1A).

We analyzed the expression of HLA-class Ib immunomodulatory molecules at the protein level on the surface of intact hAEC by flow cytometry, including HLA-E, HLA-F and HLA-G. As shown in Figure 1, panel B, hAEC expressed high surface levels of HLA-F (MRFI ± SD; 3.1 ± 0.42) and HLA-G (MRFI ± SD; 12.9 ± 1.97), whereas HLA-E surface expression was very low (MRFI ± SD; 1.35 ± 0.13). We performed analysis on 17 different batches of primary hAEC, and all the batches presented a surface profile similar to the one represented in Figure 1, panel C.

**Figure 2:**
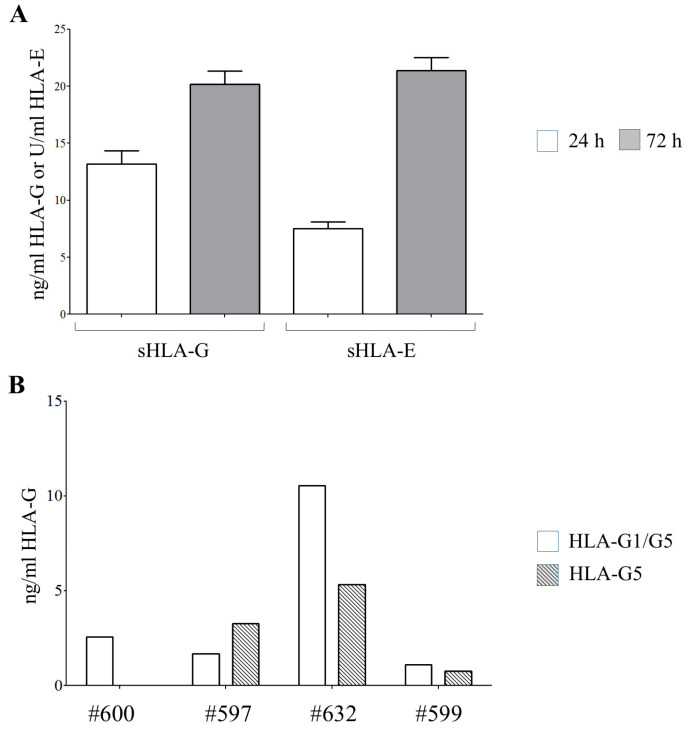
Release of HLA-class Ib molecules by hAECs. (**A**) Soluble (s) HLA-G and -E molecules were quantified by ELISA in supernatants from 6 different batches of hAECs, collected after 24 h (white columns) or 72 h (grey columns) of culture. Results are expressed as ng/mL (sHLA-G) or units/mL (sHLA-E). (**B**) sHLA-G1/G5 and sHLA-G5 were quantified by ELISA in 4 random different batches of hAEC using MEM-G9 or 5A6G7 mAbs as a capture reagent, respectively.

To assess whether hAEC release HLA-G and -E, the presence of such soluble molecules was quantified in the conditioned media derived from hAEC (*n* = 6) cultured for 24 and 72 h (Figure 2A). Every batch of hAEC released both sHLA-G (ng/mL ± SE: 13.52 ± 1.16) and sHLA-E (U/mL ± SD: 7.54 ± 0.57) after 24 h of culture, with an increasing amount after 2 more days (sHLA-G: 20.16 ± 1.15; sHLA-E: 21.38 ± 1.15). Additional experiments performed on supernatants from four different hAEC batched (using specific mAbs recognizing HLA-G1/G5 or HLA-G5 as capture reagents) revealed that both sHLA-G1 and sHLA-G5 are present (ng/mL sHLA-G1/G5: 2.55, 1.67, 10.53 and 1.09; ng/mL sHLA-G5: 0, 3.25, 5.31 and 0.77).

### 3.2. hAEC-Derived EV Expressed HLA-G and Epithelial Markers

**Figure 3:**
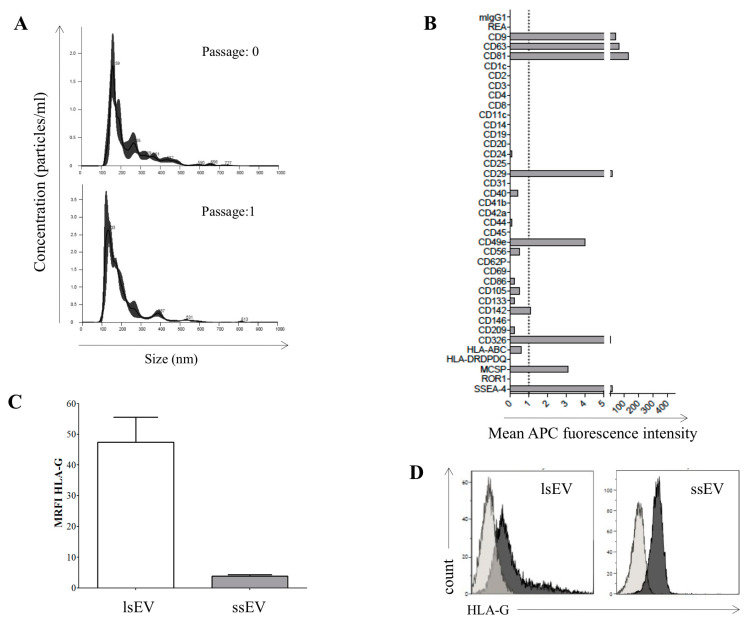
Expression of the membrane bound HLA-IbonhAEC-derived extracellular vesicle (EV). (**A**) Representative size distribution of EVs derived from 1 batch of hAEC at passage 0 (upper panel) and 1 (lower panel) and (**B**) representative multiplex-bead analysis for surface markers on small-size EV (ssEV) released by hAEC. (**C**) The expression of mHLA-G was evaluated by flow cytometry on large-size EV (lsEV) and on latex bead-conjugated ssEV isolated from 6 different batches of hAEC. Data are expressed as MRFI ± SE. (**D**) Representative staining for mHLA-G on hAEC-derived ssEV and lsEV. Light grey profiles indicate staining with isotype control antibodies, whereas dark grey profiles indicated staining with anti-HLA-G mAb.

In addition to soluble proteins, cells also release EVs that could contribute to the immunoregulatory/immunomodulatory functions of hAECs. Thus we, characterized EVs derived from hAECs next. Amnion epithelial cells generate high amounts of EVs, quantified as an average amount of 1.39 ± 0.09 × 10^9^ released particles per milliliter of medium (*n* = 3). A representative size distribution of EV derived from the conditioned media of hAEC at passage 0 (upper panel) and 1 (lower panel) is shown in Figure 3A.

The size distribution ranged from 100 to 600 nm, thus confirming the presence of both ssEV and lsEV. When we profiled the surface molecules expressed on ssEV released by hAEC, we detected characteristic epithelial markers, such as CD326 (EpCAM) and CD29 (beta1-integrin subunit; Figure 3, panel B), commonly described at the cellular level on hAEC [1]. We also detected molecules commonly expressed on pluripotent stem cells and previously identified on hAEC, such as SSEA-4 (Figure 3, panel B).

We further determined whether HLA-G molecules measured in hAEC-preconditioned medium were present in a soluble form or expressed on the surface of lsEV and ssEV. To address this issue, we purified EV from different batches of primary hAEC kept in culture for a few days, and we measured HLA-G presence on lsEV and ssEV by flow cytometry. As shown in Figure 3C, HLA-G was expressed at high levels on lsEV (MRFI ± SD; 47.4 ± 19.9) and, to a lesser extent, on ssEV (MRFI ± SD; 3.8 ± 0.86). The antigen density of the surface of lsEV resembled the level of expression commonly measured on intact hAEC cells (a representative staining is shown in histograms in Figure 3D).

### 3.3. HLA-G is Involved in hAEC-Mediated Inhibition of Allogeneic T Cell Proliferation

**Figure 4:**
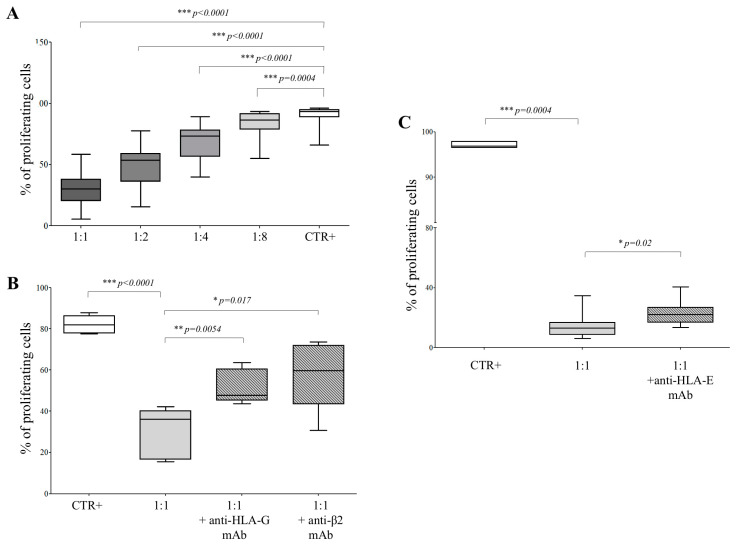
Cell proliferation assay on T cells exposed to hAEC. (**A**) CD4^+^ T cells stimulated with anti-CD3/CD28 beads (white box) were cocultured with hAEC at hAEC:T cell ratios ranging from 1:1 to 1:8 (grey boxes). Results are expressed as % of proliferating cells. Mean of 39 experiments ± SD is shown. *p* values are indicated where differences are statistically significant. (**B**) CD4^+^ T cells stimulated with anti-CD3/CD28 beads (white bar) were cocultured with hAEC at the hAEC:T cell ratio of 1:1, in the presence (grey boxes) or absence (light grey box) of anti-HLA-G blocking mAb or anti-β2 microglobulin mAb. Results are expressed as % of proliferating cells. Mean of 5 experiments ± SD is shown. *p* values are indicated where differences are statistically significant. (**C**) CD4^+^ T cells stimulated with anti-CD3/CD28 beads (white bar) were cocultured with hAEC at the hAEC:T cell ratio of 1:1, in the presence (grey boxes) or absence (light grey box) of anti-HLA-E blocking mAb. Results are expressed as % of proliferating cells. The mean of 9 experiments ± SD is shown. *p* values are indicated where differences are statistically significant.

To further understand which factors contribute to the immunosuppressive function of hAEC, we performed functional assays next. hAECs were cocultured with immune effector cells (CD4^+^ T cells) at different effector:target ratios. The proliferation rate of CD4^+^ T cells, stimulated with beads coated with anti-CD3/CD28 antibodies, was significantly reduced in the presence of hAEC, at 1:1 (% of the proliferating cells ± SD: 29.4 ± 13.2, *p* < 0.0001), 1:2 (49.4 ± 16.4, *p* < 0.0001), 1:4 (69.2 ± 14.1, *p* < 0.0001) and 1:8 (83.7 ± 9.5, *p* = 0.0004) hAEC:CD4 ratios, compared to control T cells (88.4 ± 9.9; Figure 4, panel A).

The inhibitory effect of intact hAEC was significantly reduced when such cells were pre-exposed to blocking antibodies against HLA-G or β2 microglobulin. We measured T cell proliferation side by side in T cells only (82.1 ± 4.4), and in the presence of amnion cells (1:1 hAEC:CD4 ratio) preincubated with anti-HLA-G (51.8 ± 8.2, *p* = 0.0054) or anti-β2 microglobulin (58.8 ± 17.0, *p* = 0.017) blocking antibodies, and in the absence of blocking agents (29.9 ± 12.3; *p* < 0.0001; Figure 4, panel B). Additional experiments were performed to dissect the role of HLA-E (Figure 4, panel C). Again, the percentage of proliferating T cells (97.1 ± 0.62) was significantly reduced at the 1:1 (hAEC:CD4) ratio (14.5 ± 2.8; *p* = 0.0004). The T cell proliferation was partially but significantly restored in the presence of anti-HLA-E blocking mAb (23.2 ± 2.7; *p* = 0.02), although such an effect was limited as compared to anti-HLA-G blocking mAb. These experiments revealed that both HLA-G and, to a lesser extent, HLA-E were at least in part involved in immunomodulatory activities performed by hAEC.

**Figure 5:**
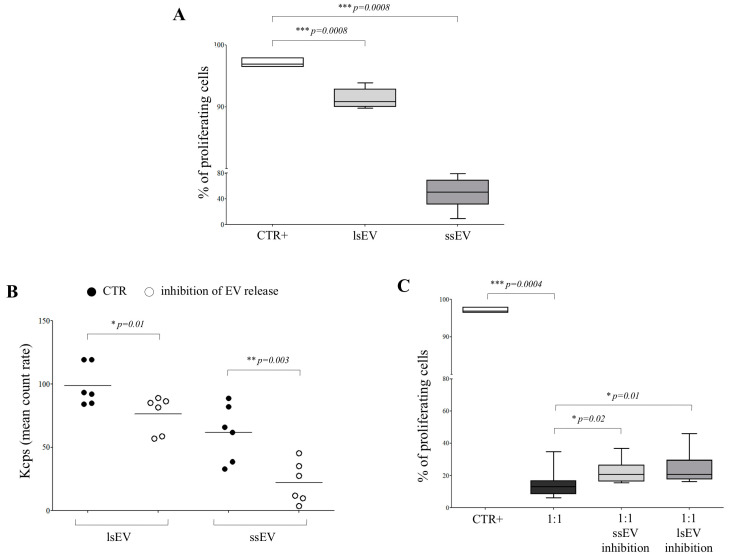
Cell proliferation assay on T cells exposed to hAEC-derived EV. (**A**) CD4^+^ T cells stimulated with anti-CD3/CD28 beads (white box) were cocultured with hAEC-derived lsEV (light grey box) or ssEV (grey box). Results are expressed as % of proliferating cells. Mean of 9 experiments ± SD is shown. *p* values are indicated where differences are statistically significant. (**B**) Concentration of lsEV and ssEV was assessed in supernatants from hAEC either untreated (black dots) or treated with inhibitors of ssEV or lsEV release (white dots). Results are expressed as the mean count rate (Kcps). (**C**) CD4^+^ T cells stimulated with anti-CD3/CD28 beads (white box) were cocultured with hAEC, either untreated (black box) or treated with inhibitors of ssEV (light grey box) or lsEV (grey box) release, at hAEC:T cell ratio of 1:1. Results are expressed as % of proliferating cells. Mean of 9 experiments ± SD is shown. *p* values are indicated where differences are statistically significant.

When immune effector cells were exposed to hAEC-derived EVs, T cell proliferation (97.1 ± 0.62) was significantly inhibited, either in the presence of lsEV (91.3 ± 1.6; *p* = 0.0008) or ssEV (49 ± 24.6; *p* = 0.0008; Figure 5, panel A). To further address the contribution of EV derived from hAEC to their immunosuppressive functions, we cultured the latter cells in the presence (or absence) of specific inhibitors of the release of ssEV (Manumycin A and GW4869) or lsEV (D-Pantethine) for 48 h. The concentration of ssEV and lsEV was analyzed in supernatants from treated cells. As shown in Figure 5B the concentration of both lsEV (mean count rate ± SD: 98.8 ± 16.2 vs. 76.3 ± 14.5; *p* = 0.01) and ssEV (61.7 ± 22.4 vs. 22.3 ± 16.4; *p* = 0.03) was significantly reduced by the treatment. Such cells were cocultured with CD4^+^ T cells at a 1:1 (effector:target) ratio in the presence of beads coated with CD3/CD28 mAbs. The percentage of proliferating T cells (97.1 ± 0.62) was significantly reduced in the presence of hAEC (14.5 ± 2.8; *p* = 0.0004). Such inhibition was significantly lower in the presence of hAEC treated with inhibitors of ssEV (22.3 ± 6.7; *p* = 0.02) or lsEV (24.8 ± 9.3; *p* = 0.01) release (Figure 5, panel C). Collectively, such experiments suggested that both ssEV and lsEV were partially involved in the immunomodulatory activity performed by hAEC.

## 4. Discussion

In allogeneic transplants, both as a solid organ, tissue or cell transplantation, immunosuppression of the recipient is the normal procedure [48]. During the last three decades, the improvement in immunosuppressive regimens has greatly reduced donor rejection, without completely solving acute and long-term side effects [49]. In immune-competent models, hAEC engrafted and survived, without administration of immunosuppressive drugs [7,8,9,13]. Seminal clinical transplants have been proved safe in allogenic hAEC injections, with no signs of rejection [50,51,52]. Nevertheless, the hAEC immune regulatory mechanism remains, for the most part, unknown. The amnion membrane represents an immunomodulatory system that typically sets up as a protective barrier for the fetus against the maternal immune system. Several groups have described restraining effects on immune effector cells exposed to hAEC, such as decreased peripheral blood mononuclear cell proliferation or activation, altered maturation of antigen-presenting or dendritic cells, phenotype switch from classically (M1) to alternatively activated (M2) macrophages and increased frequency of regulatory T and B cells [13,23,24,25,53,54]. Liu et al. observed a hAEC-mediated inhibition of mouse splenocyte proliferation in vitro, and an attenuation of the disease in a mouse model of multiple sclerosis, through a T-helper 2 (Th2) shift of T cells [54]. Similarly, recent evidence suggest that hAECs play a critical role in guiding the Th1/Th2 shift and Treg induction [55]. Notably, amnion-derived cells actively interact and cross-talk with innate and adaptive immune cells not only by cell-to-cell interactions but also through paracrine mediators. Recent reports describe hAEC capable of suppressing T cell proliferation and activation, through release of soluble factors or membrane-bound molecules [13,25,56].

To further elucidate and enhance hAEC use in clinical settings, we confirmed that hAECs are capable of suppressing T cell proliferation in vitro, evaluating the effects at different hAEC/T cell ratios. Since the mechanism(s) underlying these immunoregulatory properties of hAEC have been only partially elucidated [55], we focused on investigating the role of HLA-Ib molecules, particularly HLA-G. HLA-G can interact with different receptors, namely immunoglobulin-like transcript (ILT) 2, ILT4, KIR2DL4, CD160 and CD8α, which are differentially expressed on different cell populations. Upon interaction with these receptors, HLA-G can affect the function of immune effectors cells, leading to the suppression of the immune response in both physiological and pathological settings [33]. HLA-Ib molecules can bind peptides generated from the degradation of cytosolic proteins and present them to specific subpopulations of CD8+ T cells [20], like classical HLA-Ia molecules. We confirmed that membrane-bound HLA-G is expressed on the surface of cryopreserved hAEC and hAEC-derived EVs, but it is also released by hAEC as a soluble protein. We quantified the constitutive presence of sHLA-G1 and sHLA-G5 in hAEC culture. In the clinical procedure, cryopreserved cellular products are commonly used. Consequently, we focused our analysis on hAEC cells and products, such as EVs and soluble molecules, generated by cryopreserved cells, since these are the products manufactured in GMP conditions for clinical use [1].

In every cell batch tested, mHLA-G expression on intact cells was observed. Similarly, a low density presence of polymorphic HLA-ABC and a lack of HLA-DR was confirmed (data not shown). Moreover, the presence of soluble HLA-G1/G5 molecules in the supernatants of ex-vivo hAEC was observed, in line with previous studies [12,23]. In contrast with the systematic mHLA-G level in every cell batch analyzed, we measured restrained mHLA-E expression on the surface of hAEC. However, we demonstrated for the first time that hAEC are able to release sHLA-E in the absence of signals triggered by proinflammatory cytokines, in contrast with other cell populations that released sHLA-E upon activation [57].

In this line, upregulation of HLA-G expression on amnion-derived cells following exposure to interferon gamma has been previously reported [13,23]. Different studies reported that sHLA-E acts predominantly as an immunoregulatory molecule, whereas mHLA-E exerts immunoregulatory or immunostimulatory activities, depending on the receptor engaged [57,58]. Thus, the low expression of mHLA-E and the high release of the soluble counterpart in hAEC may suggest that HLA-E exerts immunosuppressive activities rather than immunostimulatory functions in these “immune-privileged” cells. Collectively, these data confirmed an important role of these perinatal stem cells in the modulation of innate and adaptive immunity [59].

We clearly proved that HLA-G is involved in hAEC-mediated inhibition of the T cell response, as previously suggested [13,23,25]. Proliferation of T cells was partially but significantly restored in the presence of a blocking antibody against HLA-G. Similar effects were obtained with a blocking antibody against β2-microglobulin. Blocking experiments performed with the anti-HLA-E antibody support the HLA-E role in the immunomodulatory activity played by hAEC. However, since a complete recovery in T cell proliferation was not obtained by blocking antibody activities, our data support the hypothesis that another immunomodulatory mechanism(s) is present and protect hAEC from immunorecognition [4,7,8,55].

A novel finding of this work is that HLA-G is present not only as a soluble moiety in supernatants from hAEC, but it is also expressed on ssEV and lsEV released from these cells. We provided a characterization of the EV surface phenotypes and confirm the amniotic origin of isolated EVs, as witnessed by the presence of epithelial markers, such as CD326 (EpCAM), CD29 and SSEA-4. We showed hAEC-derived ssEV and lsEV able to modulate T cell proliferation in coculture settings. In addition, intact hAEC treated with specific compounds inhibiting EV release were measured as less effective in inhibiting T cell proliferation, confirming that vesicles released by hAEC may modulate an immune cell response. Such results are in line with previous studies describing the important role of secretome, such as soluble mediators and EV released by primary hAEC [34,35].

## 5. Conclusions

We here showed that perinatal hAEC expressed and released HLA-Ib molecules. Moreover, we confirmed that these cells were able to suppress T cell proliferation in vitro, and that such an immunoregulatory capacity was partially mediated by both HLA-G and HLA-E. HLA-Ib molecules were also expressed on hAEC-derived EV, and might contribute, at least in part, to hAEC immunomodulatory activity. Such features should be carefully considered in support of clinical applications for allogenic hAEC products.
